# Measuring Unsafe Abortion-Related Mortality: A Systematic Review of the Existing Methods

**DOI:** 10.1371/journal.pone.0053346

**Published:** 2013-01-14

**Authors:** Caitlin Gerdts, Divya Vohra, Jennifer Ahern

**Affiliations:** 1 Advancing New Standards in Reproductive Health, University of California San Francisco, San Francisco, California, United States of America; 2 Division of Epidemiology, Berkeley School of Public Health, University of California, Berkeley, Berkeley, California, United States of America; Tehran University of Medical Sciences, (Islamic Republic of Iran)

## Abstract

**Background:**

The WHO estimates that 13% of maternal mortality is due to unsafe abortion, but challenges with measurement and data quality persist. To our knowledge, no systematic assessment of the validity of studies reporting estimates of abortion-related mortality exists.

**Study Design:**

To be included in this study, articles had to meet the following criteria: (1) published between September 1^st^, 2000-December 1^st^, 2011; (2) utilized data from a country where abortion is “considered unsafe”; (3) specified and enumerated causes of maternal death including “abortion”; (4) enumerated ≥100 maternal deaths; (5) a quantitative research study; (6) published in a peer-reviewed journal.

**Results:**

7,438 articles were initially identified. Thirty-six studies were ultimately included. Overall, studies rated “Very Good” found the highest estimates of abortion related mortality (median 16%, range 1–27.4%). Studies rated “Very Poor” found the lowest overall proportion of abortion related deaths (median: 2%, range 1.3–9.4%).

**Conclusions:**

Improvements in the quality of data collection would facilitate better understanding global abortion-related mortality. Until improved data exist, better reporting of study procedures and standardization of the definition of abortion and abortion-related mortality should be encouraged.

## Introduction

The true global burden of unsafe abortion-related mortality remains unknown. Employing the newest figures for global maternal mortality, the WHO estimates that in 2008 approximately 13% of maternal mortality worldwide, or 47,000 deaths were due to unsafe abortion. [1] Such estimates, however, are based on statistics from developing countries that are known to have unreliable data, [2] and because of the often sparse, poor quality data in countries where abortion is the least safe are, at best, thought to underestimate the true global incidence of mortality from unsafe abortion. [1]–[3].

Maternal deaths occur most often in settings where national vital registration systems are weak or non-existent. [1], [2] As such, measurement of maternal mortality relies on alternative methods of data collection; [4] estimates of all-cause maternal mortality can be derived from population-level surveys [5] or indirect estimation techniques. [6] Some recent methodological advances have been made in measurement techniques for all-cause maternal mortality, [6], [7] an issue that has received increased attention since the inclusion of a commitment to reductions in maternal mortality (reducing maternal mortality by 75% from 1990 levels by the year 2015) as a part of the Millennium Development Goals (MDGs) in the year 2000. Such improvements in the measurement of abortion related deaths, however, have been slow to develop. [8].

Cause-specific maternal mortality data, where cause of death is identified as one of the WHO specified direct or indirect obstetric causes of death, [9] can be captured through vital registration (death certificates), hospital or facility records (case notes and/or death certificates), verbal autopsy (a WHO validated tool for measuring cause-specific mortality at the community level through a structured questionnaire with family members of a recently deceased person, to assign cause of death (COD) in the absence of vital registration data), [10], [11] or Reproductive Age Mortality Studies (RAMOS), which combine vital registration data and verbal autopsy data. [12] Abortion-related mortality, a direct obstetric cause, is uniquely difficult to document for a number of reasons: 1) In countries where abortion is restricted or illegal altogether, women often seek abortion related services outside of the formal medical system; 2) In such settings, due to social and cultural stigma, and fear of legal consequences, women are often reluctant to seek medical services in the event of complications or reveal to family members the underlying cause of the complications; [13]–[20] 3) Because of legal consequences for patients and providers alike, clinicians who provide abortion-related services may be reluctant to report abortion-related deaths. [8], [14], [21].

The validity of existing estimates of unsafe abortion-related maternal mortality has been called into question, [1], [3] and the consequences of continuing to ignore measurement deficiencies in this field have real implications for the development of policy and implementation of programs that aim to reduce maternal mortality. However, to date there has been no assessment of the validity of existing studies that report estimates of the burden of abortion-related mortality with respect to the biases they may suffer from.

Our aim is to systematically review the available peer-reviewed evidence on unsafe abortion-related mortality published since the establishment of the MDGs (September, 2000). This review establishes criteria for evaluating the quality of research papers that cite estimates of abortion-related mortality, and presents a discussion of the methodological strengths and weaknesses of the current peer-reviewed evidence about abortion-related mortality.

## Materials and Methods

### Search Strategy

We followed a protocol adapted for the evaluation of observational studies from criteria established by the PRISMA statement. [22] Pubmed, Popline, Embase, Medline, and JStor were searched for studies published between September 1^st^, 2000 and December 1^st^, 2011. Although some relevant studies may have been excluded, due to the overwhelming majority of English language publications generated by the search, non-English language studies were excluded. Combinations of the following keywords were used in the search process: *abortion, induced abortion, unsafe abortion, maternal mortality, maternal death, pregnancy related death, cause of death, verbal autopsy*. Reference lists of relevant articles were reviewed for sources that may have been missed in the database search. The full, line-by-line search strategy for each database can be found in the appendix.

### Inclusion and Exclusion Criteria

To be included, articles had to meet the following criteria: (1) published after September 1^st^, 2000 and before December 1^st^, 2011; (2) conducted in or use data from a country where abortion is “considered unsafe”; (3) enumerated causes of maternal death, and specified “abortion” as one of those causes; (4) enumerated at least 100 maternal deaths from all causes; (5) a quantitative research study; (6) published in a peer-reviewed journal. The justification for each criterion is elaborated below.

We established calendar date restrictions for the search strategy (**Inclusion Criterion # 1**) to examine evidence published since the establishment of the Millenium Development Goals (MDGs). The MDGs set a specific target for the reduction of maternal mortality by 75% from 1990 levels by the year 2015, sparking interest in improved measurement of maternal mortality and an infusion of new funds for maternal mortality research.

Included studies were restricted to studies conducted in countries where abortion is “considered unsafe” **(Inclusion Criterion #2)**, using criteria developed by Adler *et al*. [23] While no international standard exists for the classification of such countries, Adler *et al* excluded regions of the world where the WHO classifies the incidence of unsafe abortion and associated deaths as “negligible”. [1] We followed the same classification system, resulting in the exclusion of studies from the AMRO A (Canada, Cuba, United States), EURO A (Andorra, Austria, Belgium, Croatia, Cyprus, Czech Republic, Denmark, Finland, France, Germany, Greece, Iceland, Ireland, Israel, Italy, Luxembourg, Malta, Monaco, Netherlands, Norway, Portugal, San Marino, Slovenia, Spain, Sweden, Switzerland, United Kingdom), and WPRO A (Australia, Brunei Darussalam, Japan, New Zealand, Singapore) regions. Studies which reported data in their text from in AMRO A, EURO A, and WPRO A were excluded, studies reporting data from all other regions of the world were considered for inclusion.

We included studies that enumerated the direct obstetric causes of maternal death in a study population (**Inclusion Criterion #3**), specified the cause “abortion”, and calculated the number and or proportion of maternal deaths that were due to abortion. Because the definition of abortion varies widely in the literature, various definitions were accepted including: clinical definitions of induced abortion and/or unsafe abortion; all definitions of induced abortion provided by the International Classification of Disease (Code #’s 632, 635–639, and 640.03). [9] There is compelling evidence to suggest that in low-resource settings, it is often difficult to distinguish between induced abortions spontaneous abortions, therefore, in much of the literature, abortion is defined as a combination of both the ICD definition of induced abortion (see above for code #’s) and the ICD definition of spontaneous abortion (ICD Code # 634). [24], [25] Given that this is an internationally accepted definition of abortion, definitions that combined induced and spontaneous abortion into one category were also accepted. Deaths from spontaneous abortion as an independent category were not included.

The sample size criterion (**Inclusion Criterion #4**) was established to ensure sufficient sample size for adequate precision of estimates of abortion related deaths and was based on the sample size calculations from past reviews of abortion-related sequelea. [23], [26].

We aimed to evaluate the current, quantitative evidence on the burden of abortion-related mortality. To that end, articles that did not consist of original, quantitative research **(Inclusion Criteria #5)** such as review articles, commentaries, opinion pieces, and case studies, were not included.

Finally, because this review is focused on evaluating the highest quality evidence available, only articles that had first undergone a peer-review process were eligible for inclusion (**Inclusion Criteria #6**).

### Rating Criteria

Studies were evaluated for quality on a scale modeled after a rubric developed by Charles et al [26] and derived from five primary criteria: 1) study design; 2) diagnostic procedures for assigning cause of death; 3) definition of abortion; 4) study reporting; 5) risk of bias (Table 1). Studies were ranked on the scale from Excellent to Very Poor. Table 2 provides the rubric for study evaluation.

**Table 1:** Evaluation Criteria for Study Rating.

Evaluation Criteria	+ (Positive Rating)	+/− (Satisfactory Rating)		−(Negative Rating)	
Study Design	Multiple sources of data were gathered/reviewedin order to identify as many maternal deathsas possible	More than one source of data was gathered/reviewed in the identification process for maternal deaths			Only one data source was gathered/reviewed in the identification process for maternal deaths
Diagnostic Procedures for COD Assignment	Diagnostic procedures followed standard international guidelines for COD assignment	A non-standard protocol was specifiedand followed			No protocol was specified
Definition of Abortion	One of the internationally accepted definitions of abortion was provided		One of the internationally accepted definitions of abortion was provided		No definition of abortion was provided
Study Reporting	All of the following conditions were met: 1) Thorough description of study design population, and facility characteristics was provided, 2) specific procedures for data collection, management, and analysis were reported, and 3) actual counts of maternal deaths and deaths by cause were reported	Two of the following conditions were met: 1) Thorough description of study design population, and facility characteristics was provided, 2) specific procedures for data collection, management, and analysis were reported, and 3) actual counts of maternal deaths and deaths by cause were reported			One or fewer of the following conditions were met: 1) Thorough description of study design population, and facility characteristics was provided, 2) specific procedures for data collection, management, and analysis were reported, and 3) actual counts of maternal deaths and deaths by cause were reported
**Risk of Bias**					
Negligible/Very Low	Multiple sources of bias were identified and minimized and/or accounted for in study design or analysis AND authors discussed limitations of data in detail and provided guidance for interpretation of bias.				
Low	Either multiple sources of bias were identified and minimized/accounted for in study design or analysis OR authors discussed limitations of data in detail and provided guidance for interpretation of bias.				
Moderate	Some bias was minimized through study design or analysis and some discussion of limitations of data and/or guidance for the interpretation of biases was provided.				
High	Little to no bias was minimized through design or analysis, and little to no discussion of limitations or biases was provided.				
Very High	No bias was identified or minimized through design or analysis and no discussion of limitations of data or biases therein was provided.				

**Table 2:** Rubric for evaluation of study quality.

Quality Level	Study Design	Diagnostic Procedures forCOD Assignment	Definition of Abortion	Study Reporting	Risk of Bias
Excellent	+	+	+	+	Negligible/Very Low
Very Good	+	+	+	+	Low
Fair	+/−	+	+	+/−	Moderate
Poor	+/−	+/−	+	−	High
Very Poor	−	−	−	−	Very High

+ indicates a “Positive” rating.

+/− indicates a “Satisfactory “rating.

− indicates a “Negative” rating.

### Methodological Considerations for Development of Evaluation Rubric

#### I. Sources of mortality data

Nearly two-thirds of the worlds’ countries do not routinely register vital events and thus lack complete information about births and deaths. [5], [27] Maternal mortality is often more difficult to measure than other deaths due to unique challenges in identifying and classifying maternal deaths, and especially abortion-related deaths. [5], [7] Facility-based maternal deaths are often not classified as maternal deaths if women were not registered in the labor and delivery wards (for example, the death occurred in the emergency department), and can be missed if women are not identified as pregnant, which is more likely in case of abortion-related deaths because there may not be evidence of the pregnancy, or because of reporting errors due to legal concerns about treating patients with abortion related complications. [2], [3], [28] Despite the incomplete nature of the data, maternal mortality data in low-income countries can be extracted from numerous sources including medical-facility records, vital registries (when available), coroners’ records, churches, and community registries. For community-based studies to gather the most complete possible count of maternal deaths, multiple sources of data (facility records, and community-based sources) must be reviewed to identify of maternal deaths. [28], [29] For facility-based studies, records from multiple departments or wards must be reviewed to ensure comprehensive capture of maternal deaths in the facilities. [28], [29].

#### II. Study protocol

Variations in protocol used to assign cause of death for maternal deaths are common, and the quality of data sources vary with regard to the quality of information available for cause of death assignment. [29] Nevertheless, standard clinical definitions of direct and indirect causes of maternal death exist, and international guidelines are provided by the International Classification of Disease. [9] Studies should provide a standardized definition of causes of maternal death, and should follow clinical or international standard protocol for cause of death attribution. Verbal autopsy studies must contend with an additional layer of complexity due to the non-clinical nature of the data collection process. Various algorithms based on ICD-10 definitions have been developed for clinicians and computer-based algorithms to assign cause of death from verbal autopsy data with the highest degree of validity possible. [30] While computer-based algorithms for cause of death assignment have been validated in facility-based settings, [31], [32] the generalizability of such algorithms, derived from cause of death distributions in facilities, may be limited in community settings. [33] Studies that assign cause of death from verbal autopsy data should establish the procedure used and should justify the choice of algorithm based on the study sample.

#### III. Selection bias

When the aim of a study is to document the total and cause specific burden of maternal mortality for a general population (e.g. a city, a country), facility based studies may suffer from selection bias because women with abortion related complications face a range of barriers to the access of medical services, including regulations that restrict access to safe abortion, cultural practices that stigmatize abortion, and socio-economic conditions that often lead women to attempt unsafe abortion even in settings where abortion is safely available. Facility-based data from developing countries where access to health facilities may be limited by social, cultural, and economic factors, are rarely generalizable to populations outside of those seeking medical care in facilities. Nevertheless, studies often attempt to make inference from facility-based data to a larger target population (e.g., surrounding communities). Such interpretations compromise the internal validity of facility-based studies.

The obstacles to medical care for women who have abortions outside of the formal medical system may produce underestimates of abortion-related mortality in facility-based datasets. In some circumstances, selection bias could also cause over-estimation of abortion-related mortality; in facility-based studies that use datasets collected from referral hospitals, abortion-related deaths may be over-represented as a proportion of maternal deaths. This is because a) the most severe cases may get sent directly to referral hospitals and b) delays in seeking care may disproportionately affect women with abortion-related complications resulting in those cases arriving at referral facilities too late to save the women’s lives. [25].

#### IV. Misclassification

Some women who experience complications from an unsafe abortion will seek care in health facilities; however, even among those who do, in settings where abortion is legally restricted or culturally stigmatized, women are often reluctant to disclose attempted abortion to providers. Such underreporting of abortion-related complications in facilities is a form of misclassification that almost surely leads to an underestimate of abortion related deaths. [1], [8] Verbal autopsy may provide some advantages over facility-based estimates in providing estimates of abortion related death at the community level, but the stigmatization of abortion often influences what information is reported by relatives, and may lead to misclassification. Mortality resulting from unsafe abortion is often a highly stigmatized event [13], [34] and the social, economic, and legal considerations surrounding abortion may lead to a reluctance among family members report abortion-related deaths. [20], [21].

Women who experience complications from unsafe abortion most often present to facilities with symptoms much akin to hemorrhage or sepsis. Physicians who assign cause of death may unintentionally misclassify abortion related deaths as death from hemorrhage, sepsis, or spontaneous abortion. [4], [19], [25] The risk of misclassification is heightened with verbal autopsy data, as physicians do not have the advantage of examining a patient and must rely on the accuracy of symptoms and contributing factors reported by non-clinicians. [35]–[37] Additionally, in settings where abortion is legally restricted, providers can face legal action if they provide medical care to a patient who has attempted to induce abortion. [8] Thus, in an effort to provide much needed care for their patients, providers may intentionally misclassify abortion-related complications and deaths, leading to differential misclassification that is almost certain to produce an underestimate of abortion related deaths. [37] Finally, when cause of death is unclear, it can be assigned as ‘unknown cause’, and evidence suggests that, because of its unique measurement challenges, abortion related death is more likely than the other obstetric causes to be classified as ‘unknown’. [37], [38].

All studies were evaluated with respect to the degree to which they achieved the five criteria outlined in Table 1. Emphasis was placed on the potential of study results to suffer from the various bias considerations outlined above, and the extent to which authors addressed these biases in analysis or interpretation of their findings. In addition, 10 studies were selected randomly and were reviewed by a second reviewer (DV) to determine inter-rater reliability. All studies were evaluated using the same rubric and with particular attention to the methodological considerations outlined above.

## Results

**Figure 1:**
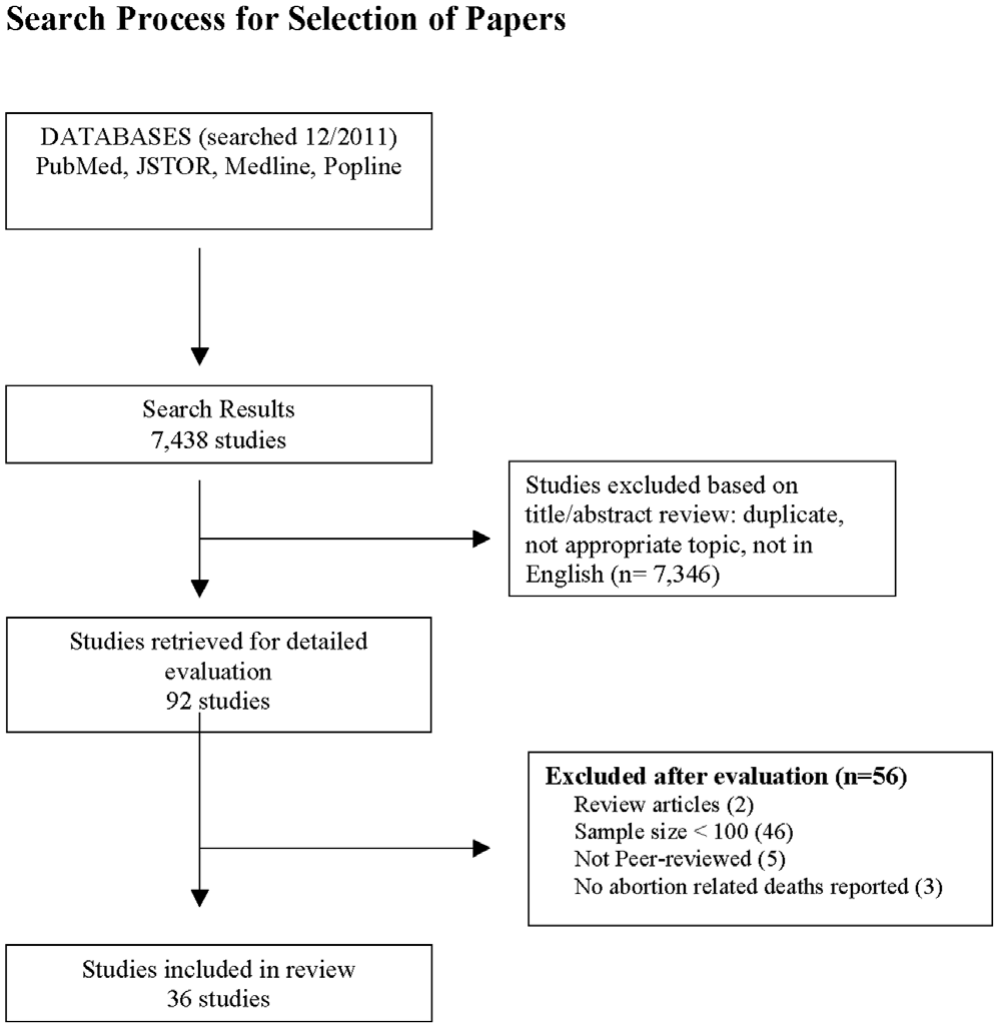
Depicts the search strategy for this review. 7,438 articles were initially identified. After excluding all duplicate titles, and reviewing titles and abstracts, the full text of 92 articles were reviewed for possible inclusion. Of those articles whose full text was reviewed, 56 did not meet inclusion criteria. The total number of studies included in the review was thirty-six.

Figure 1 summarizes the results of the search process. The initial search strategy identified 7,438 articles. After excluding all duplicate titles, and reviewing titles and abstracts for English language and relevance to the research question, the full text of 92 articles were reviewed for possible inclusion in the study. Of those articles whose full text was reviewed, 56 did not meet inclusion criteria. Two articles were review articles, synthesizing data from a variety of sources, forty-five articles did not meet the sample size inclusion criteria, five articles were not published in peer-reviewed journals, and three articles did not report any abortion related deaths in their sample. The total number of studies included in the review was thirty-six.

The thirty-six articles included in this review were conducted in a wide range of settings; the majority were conducted in Sub-Saharan Africa (n = 18), nearly one third of studies were conducted in Asia (n = 10), while four studies were conducted in Latin America and the Caribbean, and another four studies conducted in the Middle East. The articles can be divided into two types of studies: 1) *facility-based studies* (n = 22) where all data were collected at hospitals or medical facilities, and 2) *community-based studies* (n = 14) where data were collected from a variety of data sources in the community. Of the community-based studies, some included data from facilities (n = 8). A variety of study designs were used, not all of which conform to traditional epidemiologic designs. However, of thirty-six included studies, twenty three retrospective designs, three were ambi-directional designs, and ten were prospective designs. Sample sizes of the included studies ranged from 104–769 maternal deaths. Twenty-two out of thirty-six (61.1%) studies provided a clinical or international standard definition of abortion. No study presented confidence intervals, or any measure of precision for estimates of abortion-related mortality or any other cause of mortality. Table 3 summarizes the main findings of each of the studies reviewed by quality rating.

**Table 3:** Summary of major study findings.

Study (country)	Study Design,Period of data collection	Sources of mortality data	Description of study sample, number of maternal deaths	Number of Abortion related deaths/proportion of MDs due to abortion	Risk of Bias
**Quality Rating: Very Good**					
Zakariah, et al. 2009 [45]. (Accra, Ghana)	RAMOS. One year (2002)	Medical records (admission and discharge books), death certificate books, death registers, mortuary logbooks and individual case notes	All maternal deaths occuring in Accra, Ghana during study period. **179** maternal deaths.	**37** abortion related deaths (**20.8%** of all maternal deaths)	**Low**. It is possible that some deaths that were not reported to any of the multiple data sources for mortality, were not recorded by the study.
Ramos, et al. 2007 [46]. (Argentina)	Multi-center, population based, prospective study using RAMOS. One year (2002)	Hospital/clinical records, discharge records, death certificates, verbal autopsies	All maternal deaths occuring in 5 provinces with highest MMR in Argentina during the study period. **121** maternal deaths.	**33** abortion related deaths (**27.4%** of all maternal deaths)	**Moderate.** Despite the authors best efforts, because of the highly restrictive status of abortion in Argentina, it is likely that some under-reporting of abortion related deaths may be reflected in the data, and there is no discussion of potential biases or misclassification that might be involved in this analysis.
McCaw-Binns, et al. 2008 [47]. (Jamaica)	Monthly active surveillance to identify and document the deaths of all women between 15 and 49 years of age, with evidence of pregnancy. Six years (1998–2003).	Admission registers for all hospital morgues and wards, discussions with health care providers, local district registrars, funeral homes, parish police headquarters, traditional birth attendants records	All maternal deaths occuring in Jamaica during study period. maternal deaths. **540** maternal deaths	**24** aboriton related deaths (**4.4%** of all maternal deaths)	**Moderate**. Despite the authors herculean data collection efforts, as they acknowledge,abortion related deaths are likely underreported and/or misclassified.
McCaw-Binns, et al. 2007 [48]. (Jamaica)	Active surveillance of maternal deaths in public hospitals in Jamaica. Six years (1998–2003).	Institutional death and casualty registers and delivery books, case notes	All maternal deaths occuring in public hospitals in Jamaica during the study period. **232** maternal deaths.	**13** abortion related deaths (**5.6%** of all maternal deaths)	**Moderate.** Despite the authors confidence that nearly all (if not all) maternal deaths in public facilities were indeed captured, because of the sensitive nature of abortion, it is likely that aboriton related deaths are under-reported and/or misclassified.
Sloan, et al. 2001 [49].(Mexico)	Re-analysis of verbal autopsy study conducted on all reported maternal deaths in three Mexican states. One year (1995).	Verbal autopsy, all known sources of death records, death certificates, additional follow-up verbal autopsy.	All maternal deaths reported over the study period. 145 maternal deaths.	Various analytic methods used, revealed a range of **1.4–5.5%** of all maternal deaths	**Moderate.** Despite the authors best attempts and their acknowledgement of the limitations of their data, there is still the distinct possibility that these data have been biased by self-report and the lack of standardization in attribution of COD.
Chowdhury, et al. 2007 [50]. (Matlab, Bangladesh)	Cohort study. Thirty years (1976–2005)	Monthly household visit semi-structured questionnaire with relatives of all women who died when aged 15–49 years, verbal autopsy, routine demographic surveillance.	All maternal deaths identified in two neighboring areas in Matlab during the 30 year cohort study. **769** maternal deaths.	**143** abortion related deaths (**18.6%** of all maternal deaths)	**Low-Moderate.** Despite the exceptional data collection processes and the extensive interviewer training program, even under such ideal circumstances the authors note that some misclassification may have occurred.
Chowdhury, et al. 2009 [51]. (Matlab, Bangladesh)	Retrospective review of multiple sources of secondary data. Thirty years broken into three, 10-year intervals (1976–1985, 1986–1995, 1996–2005) for two areas of Matlab, Bangladesh where services are provided by ICDDRB and Government Administration respectively.	Health and Demographic Surveillance System data, pregnancy- monitoring cards, facility records for pregnancy and delivery care at the Matlab Hospital and sub- centres, verbal autopsies, the Matlab Health and Socioeconomic Survey (1996), and periodical socio- economic censuses (1982, 1996, and 2005). Verbal autopsies conducted during 1976–2005 under the HDSS, special maternal death reviews to validate maternal deaths.	All maternal deaths identified from all sources of data during study period. *1976–1985, ICDDRB*: **413** MDs. *1976–1985, Gov:* **479** MDs. *1986–1995, ICDDRB:* **342** MDs. *1986–1995, Gov*: **386** MDs. *1996–2005, ICDDRB:* **180** MDs. *1996–2005, Gov.:* **279** MDs.	1976–1985, ICDDRB: **97** Abortion related deaths (ARDs) (**23.5%** of all MDs). 1976–1985, Gov: **81** ARDs (**17%** of all MDs). 1986–1995, ICDDRB: **59** ARDs (**17.3%** of all MDs). 1986–1995, Gov: 85 ARDs (**22%** of all MDs). 1996–2005, ICDDRB: **20** ARDs (**11.1%** of all MDs). 1996–2005, Gov.: **42** ARDs (**15%** of all MDs).	**Low-Moderate.** The authors offer an excellent discussion of the role of Menstrual Regulation in the decline of abortion related mortality, including increased access to contraceptives, increasing safety of MR, and increasing education of women. Nevertheless, there may still be some misclassification or underreporting of abortion related deaths, especially earlier on in the data.
Zakariah, et al. 2006 [52]. (Accra, Ghana)	Retrospective review of deaths in 4 main hospitals. One year (2000).	Three sources of hospital records were used: admission and discharge (A&D) books, mortuary pathology records, and hospital death certificate books. All death registers from 12 of the 13 registries in the Greater Accra region, retrospective reproductive age maternal mortality survey was done in the four major hospitals.	All maternal deaths identified from all sources of data during the study period. **148** maternal deaths.	**42** abortion related deaths (**17.6%** of all maternal deaths).	**Moderate.** Despite the exceptional data collection processes and the extensive efforts to capture ALL deaths in facilities, the authors note that 86% of deaths in Accra occur in facilities. The results could suffer from some selection bias with relationship to the general population of Accra, additionally the facility data themselves may suffer from misclassification and/or underreporting of such deaths.
Bartlett, et al. 2005 [53].(Afghanistan)	Two phase, retrospective, nationally representative cohort study. Three years (1999–2002).	Death identification survey, verbal autopsy.	All maternal deaths identified for which verbal autopsies could be conducted due to safety concerns. **133** maternal deaths.	**1** abortion related death (**1%** of all maternal deaths)	**High.** Despite the nationally representative nature of these data, and despite the rigor used in interviewing, and classification of death, due to the insecurity of the region interviewers were not able to conduct VA on all identified MDs, and could not report differences between populations. In addition, the restrictive nature of abortion in Afghanistan likely leads to underreporting and misclassification of abortion related death in these data.
Jafarey, et al. 2009 [54]. (Pakistan)	Ambi-directional cohort study. Three years (2005–2007)	Monthly reports of Lady Health Workers; health management information system (HMIS), records of public-sector hospitals, records of private hospitals, graveyards, and union councils, Special survey on community factors, and Verbal Autopsy	All maternal deaths identified from all sources of data during study period. **128** maternal deaths.	**1** abortion related death (**1%** of all maternal deaths)	**High**. Despite the authors very best efforts, abortion related deaths are significantly underrepresented and, likely because of stigma associated with abortion and restrictive legal status of abortion, is likely under-reported and/or misclassified in these data.
**Quality Rating: Fair**					
Campbell, et al. 2005 [55]. (Egypt)	Nationally representative cohort study. Two, one year studies (1992–1993, and 2000).	Verbal autopsy	All maternal deaths identified through verbal autopsy during study periods. 1992–1993∶**772** MDs. 2000∶**585** MDs.	1992–1993∶**13** abortion related deaths (**3%** of all maternal deaths). 2000∶**6** abortion related deaths (**1%** of all maternal deaths).	**High.** Despite the nationally representative nature of these data, no attempt to validate results of verbal autopsy is made, and no discussion is devoted or effort is made to correct potential misclassification or other validation issues. Due to the restrictive legal climate for abortion in Egypt, the reported figures are likely underestimates.
Geelhoed, et al. 2003 [56]. (Ghana).	Active surveillance of maternal deaths in a district Hospital. Thirteen years (1998–2003).	Medical records (deaths of females of reproductive age in labor ward, maternity ward, female ward, and emergency room)	All maternal deaths occuring over the study period (13 years) in Bekerum district Hospital. **229** maternal deaths.	**43** abortion related deaths **(18.8%** of all maternal deaths)	**Moderate**. despite the authors best efforts, some bias may still exist in the reporting of abortion-related deaths
Verma, et al. 2001 [57]. (Ludhiana, India)	Retrospective review of medical records. Ten years (1985–1995).	Hospital records of all obstetrics cases, emergency department, ICU, and general hospital death records for women of reproductive age.	All maternal deaths identified from the medical records during the study period. **116** maternal deaths.	**44** abortion related deaths (**41.9%** of all maternal deaths.)	**Moderate.** Procedures for this study are standardized well, and a reasonable attempt is made to capture all maternal deaths. However, due to the fact that these data are hospital data, the authors note that there may be an over-representation of abortion related deaths because only the most serious cases end up in facilities. Therefore the data may not be representative of the larger population, but may, infact provide a good estimate of the proportion of facility-based maternal deaths that are abortion related.
Bell, et al. 2008 [58]. (Burkina Faso)	Census. Five years (2001–2006)	Census data, verbal autopsy	All pregnancy related deaths identified through census and verbal autopsy. **385** pregnancy related deaths	abortion related deaths account for **6.5%** of all pregnancy related deaths.	**Moderate-High**. Census data have been shown to have insurmountable biases related to the accurate capture of mortality (recall bias) and especially maternal mortality. Additionally, despite the authors best efforts to identify some of the limitations of this study, biases surrounding the reporting of abortion and abortion related deaths, nevertheless exist.
Oyieke, et al. 2006 [59]. (Nairobi, Kenya)	Retrospective review of medical records. Five years (1995–1999).	Case notes of all maternal deaths during pregnancy, delivery and the puerperium, records in the statistics section of the hospital records department	All maternal deaths identified during the study period. **203** maternal deaths.	**53** abortion related deaths (**25.6%** of all maternal deaths)	**Moderate-High.** The high incidence of abortion related deaths may indicate the role of selection bias due to the referral nature of the hospital. Additionally, there is 20% loss to follow up in the record review, and no description is offered of characteristics of those lost to follow-up or comparison with those in the study, the reader therefore cannot assess whether biases are present.
Mogobe, et al. 2007 [60]. (Botswana)	Retrospective review of National Maternal Mortality Audit Commit- tee analyses. Two years (2004–2005).	Confidential Maternal Death Notification Form	All maternal deaths identified in facilities in Botswana during the study period. **116** maternal deaths.	**4** abortion related deaths (**3%** of all maternal deaths)	**High**. Facility based data are unlikely to be representative of the population of Botswana, which appears to be the target population for the study. In additoin, abortion comprises 3% of maternal deaths identified, a dramatic reduction from other figures for the region, indicating the presence of underreporting and/or misclassification with respect to abortion related deaths.
**Quality Rating: Poor**					
Aboyeji, et al. 2007 [61]. (Ilorin, Nigeria)	Retrospective review of medical records. Six years (1997–2002).	Hospital records of maternal deaths (unspecified sources)	All maternal deaths identified from medical records during the study period. **108** maternal deaths.	**16** abortion related deaths (**14.8%** of all maternal deaths).	**High**. Diagnostic criteria for assignment of cause of death are not provided, no attempt to account for deaths that might have been missed because of retrospective nature of data collection, no discussion of or attempt to account for the highly restrictive legal status of abortion or potential misclassification/under-reporting this might produce. Estimates likely biased.
Fabamwo, et al. 2009 [62]. (Ikeja, Nigeria)	Two designs reported: Prospective cohort and Case-Control. Three years (2000–2003)	All admitted cases of induced abortion confirmed by the patients or accompanying relations and carried out in other facilities and with specific complications.	All maternal deaths identified from all admited cases of induced abortion during the study period. **158** maternal deaths.	**39** abortion related deaths (**24.7%** of all maternal deaths).	**Moderate.** Because this study sought to capture all abortion related complications, bias relating to deaths from induced abortion is minimized. However, due to the restrictive nature of abortion in Nigeria, some underreporting and/or misclassification could still be present in the data. Additionally, because this is an urban, tertiary-care hospital, these data may not be representative of the general population which the hosptial serves (because the most serious cases are referred to this hospital, data may even reflect overestimates).
Panchabhai, et al. 2009 [63]. (India)	Retrospective review of medical records and autopsy record. Eight years (1998–2006)	Hospital medical records (unspecified sources), pathology department autopsy reports.	All cases of maternal deaths autopsied by the pathology department during the study period. **277** maternal deaths.	17 abortion related deaths (**6.1%** of all maternal deaths)	**Moderate-High**. The dataset includes only those cases for which autopsy was performed (just over half of all maternal deaths observed during the study period), this could significantly bias the data with regard to distribution of cause of death, and since no explanation is provided of the reasons for non-autopsy, and no effort to compare those maternal deaths that were excluded with those that were included, it is impossible to evaluate whether the data are biased.
Granja, et al. 2001 [64]. (Mozambique)	Retrospective review of medical records. Five years (1989–1993).	Medical records, antenatal records if available, and autopsy records	All maternal deaths identified during the study period. **239** maternal deaths.	**18** abortion related deaths (**7.5%** of all maternal deaths)	**High**. Some discrepancies were found with respect to the distribution of causes between this study and others from Mozambique, no explanation is offered for the differences, nor is any attempt made to understand potential misclassification or underreporting for different causes of death.
Puri, et al. 2011 [65].(Delhi, India)	Retrospective review of medical records. Four years (2003–2006)	Hospital medical records (unspecified sources)	All maternal deaths recorded at Hindu Rao Hospital during the study period. **130** maternal deaths.	**6** abortion related deaths **(4.6%** of all maternal deaths).	**High.** The data collection process is poorly described, there is no understanding about what the target population is indended to be, and no discussion about potential biases present in the review, collection, or assessment of the medical records. The low proportion of abortion related deaths, given the setting, likely indicates the presence of bias.
Mswia, et al. 2003 [40]. (Tanzania)	Prospective sentinal surveillance of maternal deaths. Eight and one half years (1992–1999).	Sentinal system mortality records, verbal autopsy	All maternal deaths identified in three discricts of Tanzania during the study period. **441** maternal deaths.	**8** abortion related deaths (**7.4%** of all maternal deaths)	**High.** Classification of maternal deaths is based on self report, there are major differences in COD that vary by site, and no explanation is offered, no discussion of the major differences between ‘induced’ and ‘spontaneous/unspecified’ across sites, no discussion of potential biases this might suggest.
Daramola, et al. 2005 [66]. (Lagos, Nigeria)	Retrospective review of medical records. Ten years (1989–1998).	Medical records including autopsy protocols, case notes	All maternal deaths identified during the study period on which sufficient clinical data and autopsy certification were present. **230** maternal deaths.	**24** abortion related deaths (**10.4%** of all maternal deaths)	**High.** There is no discussion of the differences between those who were not included in the study and those who were due to lack of clinical data or lack of autopsy, with regards to abortion related deaths this could result in serious under-reporitng, in addition to overall issues of potential underreporting and misclassification of abortion related deaths which is not discussed.
Oi, et al. 2007 [67]. (Lagos, Nigeria)	Retrospective review of abortion related complications and maternal deaths. Four years (2000–2003)	Hospital records of maternal deaths(unspecified sources)	All maternal deaths identified during the study period, all abortion related complications identified during the study period. **194** maternal deaths.	**39** abortion related deaths **(20.7%** of all maternal deaths). *Study reports 24.7% but this is inaccurate.*	High. This study was focused on quantifying abortion-related complications, and thus their search strategy first selected abortion related complications and was able to capture all deaths from abortion deaths, minimizing bias. However, documentation procedures are unclear, little known about population, and despite the advantages of the data collection process some bias (selection, underreporting, misclassification) may still exist.
Jain, et al. 2004 [68]. (India)	Retrospective review of medical records. Twelve years (1988–2002).	Hospital records of all obstetrics cases, emergency department, ICU, and general hospital death records for women of reproductive age, specific review or emergency admissions due to abortion related complications.	All maternal deaths occuring in the hospital during the study period. **545** maternal deaths.	**93** abortion related deaths (**17.1%** of all maternal deaths).	**Moderate.** Because the study was conducted in a tertiary hospital, these deaths cannot be assumed to be representative of the general population, and can only be generalized to those with the worst complications.
Abdel-Hady, et al. 2007 [69]. (Dakahlia, Egypt)	Prospective cohort study. Two year (2004–2005)	Confidential enquiry, review of the patient’s notes from public health facilities, Mansoura University Teaching Hospital, reports from doctors and nurses at private health facilities	All maternal deaths identified from all sources of data during study period. **179** maternal deaths.	**5** abortion related deaths (**2.8%** of all maternal deaths)	**Moderate-High**. concerted effort is made to capture all maternal deaths in the study area by triangulating all available data sources. In Egypt, where most women deliver in hospitals, this may be a fairly complete count. However, the authors offer no discussion of how COD was attributed (to what standard), no discussion of abortion/legal context or potential misclassification, very little is known about the population, and very little is known about the research process, thus it difficult to judge how good the data might be without more contextual information.
Bergsjo, et al. 2008 [70]. (Moshi, Tanzania)	Retrospective review of medical records. Five years (2000–2004).	Hospital medical records (unspecified sources), Case Notes, Maternal Death Registration Form.	All maternal deaths registered at Kilimanjaro Christian Medical Centre during study period. **119** maternal deaths.	**2** abortion related deaths **(1.7%** of all maternal deaths)	Moderate. This study made a concerted effort to go back through all records that may not have been properly recorded and attempt to address some of the potential bias/missing data, however, the authors acknowledge that bias from underreporting and potential misclassification may exist. Additionally, no description is given vis a vis the definition of abortion, or other COD.
Lema, et al. 2005 [71]. (Blantyre, Malawi)	Retrospective review of medical records. Two years (1999–2000).	Hopsital records from gynecological, labour, antenatal and postnatal wards, maternity, operating theatre and intensive care unit.	All maternal deaths identified during the study period. **204** maternal deaths.	**48** abortion related deaths (**23.5%** of all maternal deaths)	**High.** No definitions are provided, system used for COD attribution not described, no discussion of what data might have been missed due to the retrospective nature of data collection and/or underreporting and misclassification. Because this is a eferral hospital it is likely to see higher proportion of serious cases and no discussion is devoted to representativeness of data to population.
Almerie, et al. 2010 [72]. (Damascus, Syria)	Retrospective review of medical records. Twenty years (1989–2008).	Hospital medical records (unspecified sources)	All maternal deaths identified during the study period. **189** maternal deaths.	**4** abortion related deaths (**2.1%** of all maternal deaths)	**High**. No definition of abortion is provided, nor are criterea used to define other causes of death, no discussion of the legal status of abortion is provided, nor any potential limitations of measuring COD, no characteristics of the population, the area, or the facility are provided at all, so the quality of the data are very difficult to assess.
Supratiko, et al. 2002 [73]. (Indonesia)	District-based audit of maternal mortality.	District hospital records, Health center records, midwives reports, post-mortem interviews	All maternal deaths identified from all sources of data during study period. **130** maternal deaths.	**2** aboriton related deaths (**3.6%** of all maternal deaths)	**High.** Data-collection strategy is not adequately described. No definition of Maternal Death is provided, the assignment of COD is not described, nor is the algorithm or instrument used described at all.
**Quality Rating: Very Poor**					
Chhabra, et al. 2004 [74]. (Maharashtra, India)	Retrospective review of medical records for all maternal deaths due to hemmorhage. Twenty years (1982–2002).	Case records of all maternal deaths, specifically those due to haemorrhage at Mahatma Gandhi Institute of Medical Sciences.	All maternal deaths identified during the study period. **201** maternal deaths.	**4** abortion related deaths (**2.0%** of all maternal deaths)	**High.** No definition of maternal death is provided, no definition of abortion is provided. There is no discussion of procedure around assignment of COD or how misclassification might possibly come into play, while there is some analysis of trends over time, there is no attempt to validate the statistical significance of those trends, nor discuss biases or limitations to the data.
Iyengar, et al. 2009 [75]. (Rajasthan, India)	Cross-sectional Verbal Autopsy and Maternal mortality audit for deaths in past year. One year (2002–2003)	General information form, pregnancy-related death, death due to illness, and injury-related death, and care-seeking form.	All maternal deaths identified in 14 blocks in Rajasthan, India during the study period. **156** maternal deaths.	**2** abortion related deaths **(1.3%** of all maternal deaths)	**High**. This study attempted to use a verbal autopsy audit methodology, but the methodology is poorly described, a clear definition of maternal death is not provided, The authors do not provide a definition of abortion, or how they attempted to fully capture abortion related deaths
Ujah, et al. 2005 [76]. (Plateau State, Nigeria)	Prospective cohort study. Seventeen years (1985–2001)	Case files of all women dying in pregnancy and childbirth in the maternity unit of the hospital, interviews with relatives	All maternal deaths identified at the Jos University Teaching Hospital during the study period. **267** maternal deaths.	**30** abortion related deaths (**9.4%** of all maternal deaths).	**Moderate.** While this study collected data prospectively for 17 years, the authors provide no definition of maternal death, no definiton of abortion, and offer no discussion of the potential for missing cases, selection bias with respect to the general population, or any issues of underreporting and/or misclassification which are likely present in the data.
Okonota, et al. 2002 [77]. (Afikpo, Nigeria)	Retrospective review of medical records. Ten years (1990–1999).	Delivery, abortion, ‘born before arrival’, and maternal death registries at Mater Misericordiae Hospital	All maternal deaths identified during the study period. **104** maternal deaths.	**4** abortion related deaths 4**.8%** of all maternal deaths)	**High**. The authors provide no discussion of procedures or metrics used to identify maternal deaths or assign cause of deaths, and no discussion of abortion status of possible biases despite the marked departure from 10–25% of MM that are generally understood to be abortion related deaths in Nigeria.
Mariaga, et al. 2009 [78]. (Bauchi, Nigeria)	Prospective cohort study. Seven years (2001–2007)	Hospital medical records (unspecified sources), case notes	All maternal deaths identified at State Specialist Hospital Bauchi, during the study period. **767** maternal deaths.	**48** abortion related deaths (**6.3%** of all maternal deaths)	**High.** Despite the large sample size, no description of the data collection procedure or the cause of death assignment is provided, the distribution of COD differs fairly significantly from the distribution that other studies have reported for Nigeria.
Kullima, et al 2009 [79]. (Northern Nigeria)	Retrospective review of medical records. Five years (2003–2007).	Hospital case notes.	All maternal deaths discerned from case notes of pregnant women admitted into the medical center during the study period. **112** maternal deaths.	**7** abortion related deaths (**6.2%** of all maternal deaths).	**High.** This study has few merits. while it seems to suggest that it is a complete report of all maternal deaths that occurred in the facility over 5 years, it does nothing to substantiate those claims; no definition of maternal death is provided, no diagnostic procedures are provided with regard to the assignment of COD, no discussion of biases is given, no attempt to identify misclassification.

### Quality Rating

No study received a rating of *Excellent*; this can primarily be attributed to poor evaluation for the criterion “Risk of bias”. To be considered “Excellent”, studies would have had to empirically demonstrate (through validation studies or other methods) that their data were free from major sources of systematic error, or, in the absence of such freedom from bias, perform a quantitative analysis of the effect of potential biases present in the data (through sensitivity analyses or other bias correcting techniques). No study attempted either strategy.

Of the 10 randomly selected studies that were reviewed by two raters (DV and CG), all ten were assigned to the same rating categories by both raters. Although the individual ranking components varied slightly across reviewers, the overall ratings were identical for both reviewers.

Meta-analysis of the data from the thirty-six studies was determined to be inappropriate due to the wide variation in context, study design, and measures. Findings, however, were qualitatively analyzed to determine whether any discernable pattern emerged by quality, geographic region, or type of study with regard to the proportion of abortion-related deaths reported by each study. Overall, studies receiving a “Very Good” rating found the highest estimates of abortion related mortality (median: 16%, range 1–27.4%). Studies receiving a “Very Poor” rating found the lowest overall proportion of abortion related deaths (median: 2%, range 1.3–9.4%). Table 4 shows the studies by quality level and proportion of abortion related deaths reported.

**Table 4:** Proportion of abortion related deaths reported by study quality.

Rating (n)	MedianProportion	Range of Proportions Reported
Excellent	–	–
Very Good (10)	16	1–27.4
Fair (6)	6.5	1–41.9
Poor (14)	7.45	1.7–24.7
Very Poor (6)	2	1.3–9.4

Ten of thirty-six studies received the rating of *Very Good*. All studies in the *Very Good* category used multiple data sources to identify maternal deaths, provided the international standard definition of abortion (ICD version 9 or 10), and clearly described the methods used to assign cause of death. Predominantly, studies that were categorized as *Very Good* were prospective in design. Despite the lack of quantitative bias assessment, all studies receiving a *Very Good* rating enumerated the biases thought to be present in their data, and provided a thorough discussion of potential study limitations and cautions to be taken in interpreting the results of the studies. The 2001 paper by Sloan, *et al* [39] provides a notable example of such a discussion. In this paper, the authors reanalyzed data from a verbal autopsy study conducted in three regions of rural Mexico in 1995, using multiple validated methods to determine cause of death from verbal autopsy. The paper aimed to assess variations of cause of death found through the various methods used. In their discussion, the authors discuss various limitations of verbal autopsy data, stating that

“In our rural study, many women delivered at home and the information given on death certificates was probably both incomplete and inaccurate, rarely being based on pathological examination or direct observation…”.

Additionally, the authors note that variations in the distribution of cause of death using different methods for assigning cause of death were at times so great that the data became un-interpretable.

Six out of thirty-six studies received a *Fair* rating. Studies in the *Fair* category varied in the sources of data reviewed; some reviewed multiple sources of data, others reviewed only hospital records. A mix of retrospective, prospective, and ambi-directional study designs were used. All studies, however, provided a definition of abortion, and most reported with sufficient detail the procedures used to assign cause of death. No study that received a *Fair* rating provided a detailed description of limitations or the potential for biases contained in the data. One typical “Fair” study is a nationally representative cohort study of maternal deaths in Egypt, conducted by Campbell, et al in 2005. This study reviewed official records of maternal deaths, collected through active surveillance of maternal deaths during two one-year periods (1992–1993, and the year 2000) and followed up with verbal autopsy to assign cause of death. Clear definitions of maternal death and all cause of death were provided based on international standards, and the citation for ICD-10 classification of cause of death was provided. A detailed description of physician training in verbal autopsy and cause of death assignment was given, and the procedure for validation of cause of death (repeating verbal autopsies in a percentage of cases to ensure validity of initial recording) was clearly articulated. Despite the large sample size (772 maternal deaths in the first year, 585 in the second year) and the nationally representative nature of the data, the authors provide no discussion of the general limitations of verbal autopsy for assigning cause of death nor do they provide any assessment of potential misclassification or underreporting that could have occurred with respect to abortion related deaths because of the legal status or stigma surrounding abortion.

Fourteen of thirty-six studies received a rating of *Poor*. These studies predominantly used retrospective study designs, most were facility-based studies, and no studies categorized as *Poor* used multiple sources of data to identify maternal deaths. Only three studies in this category provided a definition of abortion (two studies reported clinical definitions, one study reported ICD-9 definitions), few studies offered descriptions of the protocol followed or the process used to assign cause of death, and no study provided a thorough discussion of biases and limitations of their data. Additionally, some studies in the Poor category found smaller or larger proportions of maternal death attributable to abortion than what is suggested by the general literature or other studies in a similar geographic region. When such findings occurred, studies rated *Poor* were most likely to dismiss the results of other studies, or ignore the contradiction all together. One such discrepancy can be found in the paper by Mswia et al. [40] Despite the prospective nature of the study, and the explanation of protocol used to assign cause of death, significant variations in distribution of cause of death are found across study sites. Though the authors claim that the rural sites are similar in size and socio-economic make-up, no explanation is provided about factors that might be considered as driving the differences in distribution of cause of death across sites, nor is any discussion devoted to the discrepancy between the studies’ finding of abortion related deaths (7.4% of maternal deaths) and other studies that have suggested a higher proportion (up to 20% of maternal deaths [1], [28]) in East Africa.

Six out of thirty-six studies received a rating of *Very Poor*. All studies in this category were facility-based studies, though the directionality of the study designs varied, none of the studies receiving a *Very Poor* rating used multiple sources of data to identify maternal deaths. None of these studies reported any definition of abortion, and few provided any description of the process or protocol followed in the assignment of cause of death. The discussion sections of these papers were found to be severely lacking, and most of the studies in the *Very Poor* category failed to discuss any limitations of the study or the data.

## Discussion

A few notable trends emerge with respect to the quality of studies in this systematic review. First, more than half (54%) of all studies reviewed were categorized in the lowest two possible categories of quality ratings, and not one study achieved the highest possible quality rating. Such results highlight the need for a thorough examination of data sources, data collection techniques, and study reporting in the maternal mortality literature. Second, even among studies receiving a *Very Good* rating, where maternal mortality estimates were determined to be more valid, the risk of bias in the data reported was moderate to high. While some studies acknowledged the presence of selection bias or misclassification only one study addressed potential biases by using multiple techniques in attempt to validate results [39] and not one study out of thirty-six presented any quantitative assessment of the role of potential biases on their results. Recent developments in analytic tools that allow for the evaluation of sensitivity to multiple potential sources of systematic error and bias [41]–[43], could be extremely productive when applied to estimates of abortion related mortality. Third, the majority of studies in this systematic review failed to provide a clear definition of abortion, or abortion-related mortality. Without a standard definition, it becomes nearly impossible to compare results across studies or draw conclusions regarding trends of abortion related mortality globally, regionally, or locally. Some controversy surrounding the definition does indeed exist; while the current ICD-10 standard is to separate induced abortion from spontaneous abortion [9] when measuring incidence of abortion as well as abortion-related death, some have suggested that the risk of misclassification, in both directions, indicates that induced and spontaneous abortions should be measured as one category [23]. Misclassification of induced vs. spontaneous abortion related deaths may be present in some of the studies reviewed here. It was beyond the scope of this systematic review to determine the direction or magnitude of such misclassification, however, regardless of which measure is ultimately chosen, it is imperative that the field settle on a clear and precise definition of abortion.

An additional trend emerges from the results of studies in this systematic review; on average, studies of higher quality reported estimates of abortion-related mortality that were higher than the estimates of abortion-related mortality reported by studies of lower quality. While meta analysis of the studies included in this review was not possible, this finding supports the widely stated position that current estimates of maternal mortality due to unsafe abortion, which are primarily estimated from resource poor settings where high quality data collection is most challenging, [1] are likely under-estimating the true burden of unsafe abortion-related mortality.

While many studies in the review had substantial limitations, this systematic evaluation allowed identification of key directions for improvement of future research. Improvements in the quality of data sources and data collection are the ultimate solution to better understanding global abortion-related mortality, and recent calls for investments from the global community in vital registration systems for all countries may go a long way to addressing such issues. [2], [11], [44] In the mean time, the field should encourage better reporting of study procedures and standardization of the definition of abortion and abortion-related mortality, and should support the use of multiple bias analysis techniques in the reporting of data, a method that could greatly aid the interpretation of results from studies seeking to quantify abortion related mortality.

## Supporting Information

Appendix S1 **Provides a line by line search strategy for all databases searched.**
(PDF)

PRISMA Checklist S1 (DOC)
