# Supplementary material for: Measuring Unsafe Abortion-Related Mortality: A Systematic Review of the Existing Methods
Source: PLoS One. 2013 Jan 14;8(1):e53346. doi: 10.1371/journal.pone.0053346 (PMC3544771; doi:10.1371/journal.pone.0053346)
Supplement: Appendix S1 — Provides a line by line search strategy for all databases searched. (PDF) [file pone.0053346.s001.pdf]

Appendix 1: Final search strategy

Pubmed:

1. Limit to human
2. (maternal adj4 mortal$).af.
3. (maternal adj4 death$).af.
4. (pregnan$ adj4 death$).af.
5. (pregnan$ adj4 mortalit$).af.
6. exp abortion
7. exp pregnancy termination
8. exp menstrual regulation
9. exp verbal autopsy
10. or/2-9
11. 2-4 and 6
12. 2-4 and 7
13. 2-4 and 8
14. 2-8 and 9
15. "2000".yr. and 2-9
16. "2001".yr. and 2-9
17. "2002".yr. and 2-9
18. "2003".yr. and 2-9
19. "2004".yr. and 2-9
20. "2005".yr. and 2-9
21. “2006”,yr. and 2-9
22. “2007”,yr. and 2-9
23. “2008”,yr. and 2-9
24. “2009”,yr. and 2-9
25. “2010”,yr. and 2-9
26. “2011”,yr. and 2-9

Medline (Ovid):

1. Limit to human
2. (maternal adj4 mortal$).af.
3. (maternal adj4 death$).af.
4. (pregnan$ adj4 death$).af.
5. (pregnan$ adj4 mortalit$).af.
6. exp abortion
7. exp pregnancy termination
8. exp menstrual regulation
9. exp verbal autopsy
10. or/2-9
11. 2-4 and 6
12. 2-4 and 7
13. 2-4 and 8
14. 2-8 and 9
15. "2000".yr. and 2-9
16. "2001".yr. and 2-9
17. "2002".yr. and 2-9
18. "2003".yr. and 2-9
19. "2004".yr. and 2-9
20. "2005".yr. and 2-9
21. “2006”,yr. and 2-9
22. “2007”,yr. and 2-9
23. “2008”,yr. and 2-9
24. “2009”,yr. and 2-9
25. “2010”,yr. and 2-9
26. “2011”,yr. and 2-9

EMBASE (Ovid):

1. Limit to human
2. (maternal adj4 mortal$).af.
3. (maternal adj4 death$).af.
4. (pregnan$ adj4 death$).af.
5. (pregnan$ adj4 mortalit$).af.
6. exp abortion
7. exp pregnancy termination
8. exp menstrual regulation
9. exp verbal autopsy
10. or/2-9
11. 2-4 and 6
12. 2-4 and 7
13. 2-4 and 8
14. 2-8 and 9
15. "2000".yr. and 2-9
16. "2001".yr. and 2-9
17. "2002".yr. and 2-9
18. "2003".yr. and 2-9
19. "2004".yr. and 2-9
20. "2005".yr. and 2-9
21. “2006”,yr. and 2-9
22. “2007”,yr. and 2-9
23. “2008”,yr. and 2-9
24. “2009”,yr. and 2-9
25. “2010”,yr. and 2-9
26. “2011”,yr. and 2-9

POPLINE

Under keywords:

="Maternal Mortality" / =”Maternal Death” /="Pregnancy Death" / ="Pregnancy Mortality" / ="Abortion Induced" / = “Abortion”/ = “Menstrual Regulation” /= “Verbal Autopsy”

JSTOR

Under Keywords:
="Maternal Mortality" / =”Maternal Death” /="Pregnancy Death" / ="Pregnancy Mortality" / ="Abortion Induced" / = “Abortion”/ = “Menstrual Regulation” /= “Verbal Autopsy”
